# Structural Analysis of Papain-Like NlpC/P60 Superfamily Enzymes with a Circularly Permuted Topology Reveals Potential Lipid Binding Sites

**DOI:** 10.1371/journal.pone.0022013

**Published:** 2011-07-22

**Authors:** Qingping Xu, Neil D. Rawlings, Hsiu-Ju Chiu, Lukasz Jaroszewski, Heath E. Klock, Mark W. Knuth, Mitchell D. Miller, Marc-Andre Elsliger, Ashley M. Deacon, Adam Godzik, Scott A. Lesley, Ian A. Wilson

**Affiliations:** 1 Joint Center for Structural Genomics, La Jolla, California, United States of America; 2 Stanford Synchrotron Radiation Lightsource, SLAC National Accelerator Laboratory, Menlo Park, California, United States of America; 3 Wellcome Trust Sanger Institute, Wellcome Trust Genome Campus, Hinxton, Cambridgeshire, United Kingdom; 4 Center for Research in Biological Systems, University of California San Diego, La Jolla, California, United States of America; 5 Program on Bioinformatics and Systems Biology, Sanford-Burnham Medical Research Institute, La Jolla, California, United States of America; 6 Protein Sciences Department, Genomics Institute of the Novartis Research Foundation, San Diego, California, United States of America; 7 Department of Molecular Biology, The Scripps Research Institute, La Jolla, California, United States of America; Weizmann Institute of Science, Israel

## Abstract

NlpC/P60 superfamily papain-like enzymes play important roles in all kingdoms of life. Two members of this superfamily, LRAT-like and YaeF/YiiX-like families, were predicted to contain a catalytic domain that is circularly permuted such that the catalytic cysteine is located near the C-terminus, instead of at the N-terminus. These permuted enzymes are widespread in virus, pathogenic bacteria, and eukaryotes. We determined the crystal structure of a member of the YaeF/YiiX-like family from *Bacillus cereus* in complex with lysine. The structure, which adopts a ligand-induced, “closed” conformation, confirms the circular permutation of catalytic residues. A comparative analysis of other related protein structures within the NlpC/P60 superfamily is presented. Permutated NlpC/P60 enzymes contain a similar conserved core and arrangement of catalytic residues, including a Cys/His-containing triad and an additional conserved tyrosine. More surprisingly, permuted enzymes have a hydrophobic S1 binding pocket that is distinct from previously characterized enzymes in the family, indicative of novel substrate specificity. Further analysis of a structural homolog, YiiX (PDB 2if6) identified a fatty acid in the conserved hydrophobic pocket, thus providing additional insights into possible function of these novel enzymes.

## Introduction

NlpC/P60 superfamily proteins [1] are ubiquitous papain-like cysteine peptidases or other functionally related enzymes. Characterized members of this superfamily have diverse enzymatic functions, such as peptidases, amidases, transglutaminases and acetyltransferases. Detailed sequence analysis [1] suggested that this divergent superfamily consists of four main families: P60-like, AcmB/LytN-like, YaeF/YiiX-like, and LRAT-like. P60-like and AcmB/LytN-like enzymes are hydrolases with specificity for amide linkages in cell-wall components, such as those in D-γ-glutamyl-*meso*-diaminopimelate and N-acetylmuramate-L-alanine. These two families are **c**anonical papain-like NlpC/P60 enzymes (**C**PNEs) with a catalytic core similar to that of papain, which has been confirmed by structural studies [2], [3], [4], [5]. The latter two families were predicted to contain a conserved catalytic triad (Cys, His and a polar third residue) in a circularly permuted catalytic domain where the relative positions of the cysteine and histidine/polar residue are swapped in the primary sequence [1], which we will refer to as **p**ermuted papain-like NlpC/P60 enzymes (**P**PNEs).

PPNEs are involved in a number of important processes. YaeF/YiiX-like members are found in poxviruses and pathogenic bacteria. For example, G6R is a conserved protein that contributes to virulence of vaccinia virus [6]. Recently, it was shown that the RID (Rho GTPase inactivation domain) of *Vibrio ÿholera* MARTX toxin is also a circularly permuted papain-like cysteine peptidase [7]. These proteins are believed to be important in pathogen-host interactions and, thus, are potential candidates for drug targeting. Several characterized eukaryotic proteins also contain a PPNE domain, such as LRAT (lecithin retinol acyltransferase) [8], nematode developmental regulator Egl-26 [9], [10], and class II tumor suppressor H-rev107 [11], [12], which was recently shown to function as a thiol hydrolase-type phospholipase A1/2 [13]. Furthermore, bioinformatics studies suggested that PPNEs are related to the PPPDE (Permuted Papain fold Peptidases of DsRNA viruses and Eukaryotes) superfamily, which has a potential role in the ubiquitin signaling pathway [14].

Other than LRAT, currently little information is available on the biochemical function of PNPEs. A subset of structural genomics projects have focused on determining structures of protein families that are largely uncharacterized, thus providing unique opportunities for studying their functions from a structural perspective. To date, three representatives of this interesting protein family have been determined by structural genomics groups. They include YiiX from *Escherichia coli* by NYSGXRC (New York SGX Research Center for Structural Genomics, PDB 2if6, unpublished results), BcPPNE (stands for *Bacillus cereus* PPNE) by the Joint Center for Structural Genomics (JCSG, PDB 3kw0, this work), and human PPPDE1 by SGC (Structural Genomics Consortium, PDB 3ebq, unpublished results).

To provide insights into the function of these biologically important proteins, as well as PPNEs in general, we report the crystal structure of BcPPNE and a comparative structural analysis to other related PPNEs. These structures clearly confirm the previous prediction of a permuted topology of the PPNEs [1]. We show that the arrangement of the PPNE catalytic residues is similar to those of CPNEs. All three PPNEs possess a hydrophobic S1 substrate-binding pocket, which differs from previously characterized CPNEs. Furthermore, we have identified ligands in the active sites of BcPPNE and YiiX, which have lead to new functional insights. Our results suggest that BcPPNE and YiiX are likely amidases with specificity for the amide bond between a lipid and an amino acid (or peptide).

## Results

### Structural determination and structural quality

BcPPNE is likely a cytoplasmic protein with a molecular weight of 22.2 kDa (residues 1–195) and a calculated isoelectric point of 5.3. The crystal structure of BcPPNE was determined using the high-throughput structural genomics pipeline implemented at the JCSG (http://www.jcsg.org) [15], [16]. The selenomethionine derivative of BcPPNE was expressed in *E. coli* with an N-terminal TEV cleavable His-tag and purified by metal affinity chromatography. The data were indexed in space group P6_5_ and the structure was determined to a resolution of 2.5 Å with four molecules per asymmetric unit (asu) using the SAD method (R_cryst_ = 19.2/R_free_ = 21.9). The mean residual error of the coordinates was estimated to be 0.25 Å by a diffraction-component precision index method (DPI) [17]. The electron density was well defined for the majority of the protein. The BcPPNE model displays good geometry with an all-atom clash score of 8.3 and the Ramachandran plot produced by MolProbity [18] shows that all, but three, residues are in allowed regions, with 96.7% in favored regions. The three Ramachandran outliers (B1, B170 and C170) are located in regions where the electron density is poor. The final structure of BcPPNE contains four monomers (A, residues 2–195; B, residues −3–195; C residues −4–195; and D, residues 0–195, where residues upstream of 1 are a part of the purification tag), with a lysine bound in each active site, nine chloride ions and 41 waters. Identification of residues from the N-terminal purification tag in the density maps suggested that the tag was not cleaved, in agreement with mass spectroscopy (data not shown). The crystal structure indicates that the cleavage site is not readily accessible to TEV since it is located at the start of a helix. Sections of the 171–175 loop, the N-terminal purification tag, and some side chains on the protein surface, were disordered and not included in the final model. Data collection, refinement and model statistics are summarized in Table 1.

**Table 1:** Data collection, phasing and refinement statistics for BcPPNE (PDB 3kw0).

**Space group**	P6_5_
**Unit Cell**	*a* = 65.0 Å, *c* = 407.8 Å
**Data collection**	λ_1_ SADSe (peak)
Wavelength (Å)	0.9794
Resolution range (Å)	37.8–2.5
Number of observations	190,686
Number of unique reflections	33,303
Completeness (%)	99.7 (99.7)^a^
Mean I/σ(I)	15.3 (1.7)^a^
R_merge_ on I (%)	8.0 (104)^a^
R_meas_ on I (%)	8.8 (114)^a^
R_pim_ on I (%)	3.7 (47.6)^a^
Highest resolution shell (Å)	2.64–2.50
**Model and refinement statistics**	
Resolution range (Å)	37.8–2.5
No. reflections (total)	33,302
No. reflections (test)	1,689
Completeness (% total)	99.7
Cutoff criteria	|F|>0
R_cryst_ (%)	19.2
R_free_ (%)	21.9
**Stereochemical parameters**	
Restraints (RMS observed)	
Bond lengths (Å)	0.014
Bond angles (°)	1.5
Average isotropic B-value	
Overall (Å^2^)	66.2
Ligand (Å^2^)	51.3
ESU based on R_free_ (Å)	0.25
Protein residues/atoms	773/6,013

a Highest resolution shell in parentheses. The high resolution cutoff was chosen such that the mean I/σ(I) in the highest resolution shell is around 2. These statistics were calculated assuming the equivalence of Friedel pairs.

ESU = Estimated Standard Uncertainty in atomic coordinates.

R_merge_ = Σ_hkl_ Σ_i_|I_i_(hkl)−<I(hkl)>|/Σ_hkl_Σ_i_I_i_(hkl), R_meas_ (redundancy-independent R_merge_) = Σ_hkl_[N_hkl_/(N_hkl_−1)]^1/2^ Σ_i_|I_i_(hkl)−<I(hkl)>|/Σ_hkl_ Σ_i_I_i_(hkl), and R_pim_ (precision-indicating R_merge_) = Σ_hkl_[1/(N_hkl_−1)]^1/2^ Σ_i_|I_i_(hkl)−<I(hkl)>|/Σ_hkl_ Σ_i_I_i_(hkl).

R_cryst_ = Σ_hkl_||F_obs_|−|F_calc_||/Σ_hkl_|F_obs_| where F_calc_ and F_obs_ are the calculated and observed structure factor amplitudes, respectively.

R_free_ = as for R_cryst_, but for 5.0% of the total reflections chosen at random and omitted from refinement.

### Structural description

**Figure 1:**
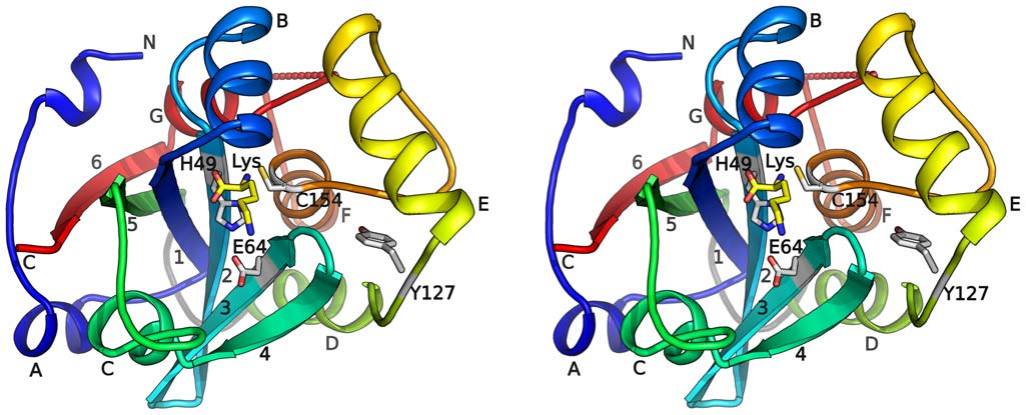
Crystal structure of BcPPNE. Stereoview of ribbon representation of BcPPNE is color coded from blue (N-terminus) to red (C-terminus). The α-helices are labeled A to G, and β-strands 1 to 6. The bound lysine and active site catalytic residues are shown as sticks. The loop that unites the circular permuted sub-domains (residues 103–110) is colored grey. The 3_10_ helices are not labeled.

The four monomers in the asu are nearly identical with an average rmsd of 0.36 Å for 188 C_α_ atoms. Each monomer consists of a layered α/β fold with a central, 6-stranded, antiparallel β-sheet (β1–β6), which is protected by helices on either side (αA and αC on one side, αD, αF and αG the other) (Fig. 1). The catalytic Cys154 is located on the N-terminus of helix αF. Two additional helices (αB and αE) are located above Cys154, contributing to the formation of the active site, where the lysine ligand is bound.

Only a few close homologs for BcPPNE (seq id > = 25%) have been identified in proteobacteria and one in eukaryotes. The closest bacteria homolog from *Tolumonas auensis* shares 50% sequence identity with BcPPNE. The eukaryotic homolog from *Plasmodium knowlesi* (seq id 27%), a major human malaria parasite, contains an N-terminal PH domain. This sporadic distribution of close BcPPNE homologs may reflect the highly divergent nature, as well as the complex evolutionary history, of the NlpC/P60 superfamily [1]. Several conserved residues are likely important for the structural integrity of the overall protein architecture and active site. Asp25, which is part of a highly conserved GD motif conserved across the NlpC/P60 superfamily, is located near the N-terminus of β1 and forms hydrogen bonds with Arg98 from the neighboring β5. Glu157 and Arg138 form a buried salt bridge, connecting αD and αF. The hydrogen bond between the Ser155 hydroxyl and the Ile177 carbonyl group is likely important for the structural integrity of the active site. Besides the strictly conserved catalytic residues (Cys154, His49, Glu64 and Tyr127), other residues near the bound lysine are also highly conserved, including Ser36, Ser48, Tyr79, Tyr83, and Tyr90.

### A common core shared by NlpC/P60 superfamily proteins

**Figure 2:**
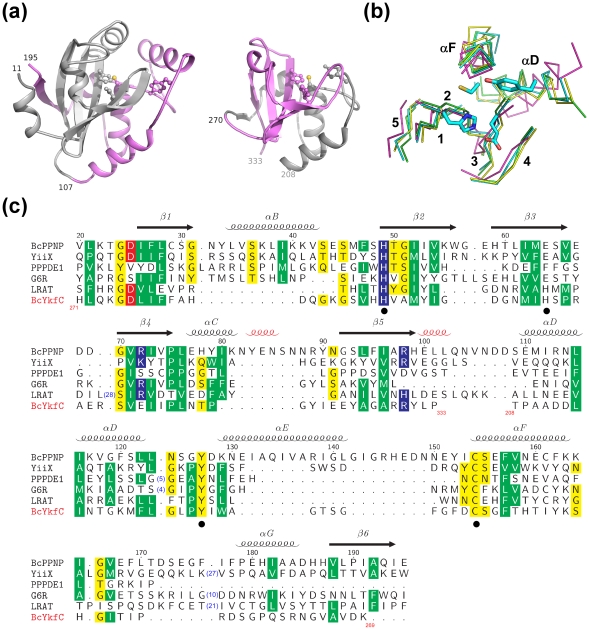
Common core of NlpC/P60 superfamily. (a) Structure comparison between BcPPNE (left) and CPNE BcYkfC (right, PDB 3h41). The N-terminal subdomains of both proteins are colored grey, and the C-terminal subdomains in violet. The catalytic residues are shown as ball-and-sticks. The sites of the circular permutation are indicated by residue numbers. (b) Core conserved secondary structures within the NlpC/P60 superfamily. C_α_ traces for the conserved cores of BcPPNE (green), YiiX (cyan), BcYkfC (yellow), PPPDE1 (magenta) the conserved residues are superposed. Secondary structures of BcPPNE (green) and catalytic residues of YiiX (sticks) are highlighted. (c) Sequence alignment between PPNEs (BcPPNE, YiiX, human PPPDE1, vaccinia virus G6R, human LRAT), and CPNE BcYkfC. Sequence numbering and secondary structures of BcPPNE are shown at the top. The 3_10_ helices are shown in red and other secondary structures are labeled as in Fig. 1. Sequences of the two subdomains of BcYkfC are swapped in comparison to the others and the respective residue ranges are shown near the ends of the permuted segments.

We have previously determined the structure of a γ-D-glutamyl-L-diamino acid endopeptidase YkfC from *B. cereus* (BcYkfC), a member of the P60-like family [5]. The catalytic domain of BcYkfC is a prototypical CPNE consisting of 126 residues (residues 208–333). The N-terminal (residues 11–107) and the C-terminal (residues 108–195) portions of BcPPNE are circularly permuted, compared to BcYkfC (Fig. 2a). The N-terminal region folds into a subdomain consisting of a 5-stranded β-sheet protected by connecting loops on one side. The C-terminal subdomain consists of mainly helices with an additional C-terminal β-strand (β6) augmenting the open edge of β5 of the N-terminal subdomain. As each subdomain contains two catalytically important residues, the circular permutation also results in swapping of the active site residues. The sites of permutation are approximately distal to the active site (Fig. 2a), which could help minimize the effect of the permutation and maintain the integrity of the active site. BcPPNE is very similar to BcYkfC with an rmsd of 1.7 Å for 97 equivalent C_α_ atoms, despite the circular permutation.

BcPPNE and YiiX are closely related with an rmsd of 2.1 Å for 146 C_α_ atoms and sequence identity of 20.5%. Comparisons with the available PPNEs and CPNEs structures allowed the identification of a structural core, which we expect to be conserved among all members of the NlpC/P60 superfamily (Fig. 2b–c). This core consists of the 5-stranded β-sheet of the N-subdomain (β1–β5) and two helices of the C-subdomain (αD and αF of BcPPNE). The functional importance of this core is clear since it houses the catalytic residues. Additionally, a short helix following αF (αG of BcPPNE) is also generally conserved and is likely to stabilize β1 and β2.

PPPDE1, a member of the PPPDE superfamily, also contains a similar core with a conserved arrangement of catalytic residues (Fig. 2b). However, some structural differences are observed within the core compared to the other two PPNEs members. PPPDE1 can be superposed onto PPNE YiiX with an rmsd of 4.2 Å for 119 C_α_ atoms, and onto CPNE BcYkfC with an rmsd of 3.2 Å for 97 C_α_ atoms, compared to an rmsd of 2.4 Å for 119 C_α_ atoms for PPNE YiiX with CPNE BcYkfC. Thus, the PPPDE1 structure supports the unification of the PPPDE and PPNE superfamilies, even although the sequence similarity between these proteins is very weak (Fig. 2c). The spatial location of the C-terminal portion of PPPDE1 (after the helix containing the catalytic cysteine) is significantly different from other NlpC/P60 proteins. This region forms two helices that contribute to the formation of a S1 substrate-binding pocket. In contrast, the equivalent region in BcPPNE (αG-β6) and other structures augments the conserved core.

### A conserved active site with a bound lysine

**Figure 3:**
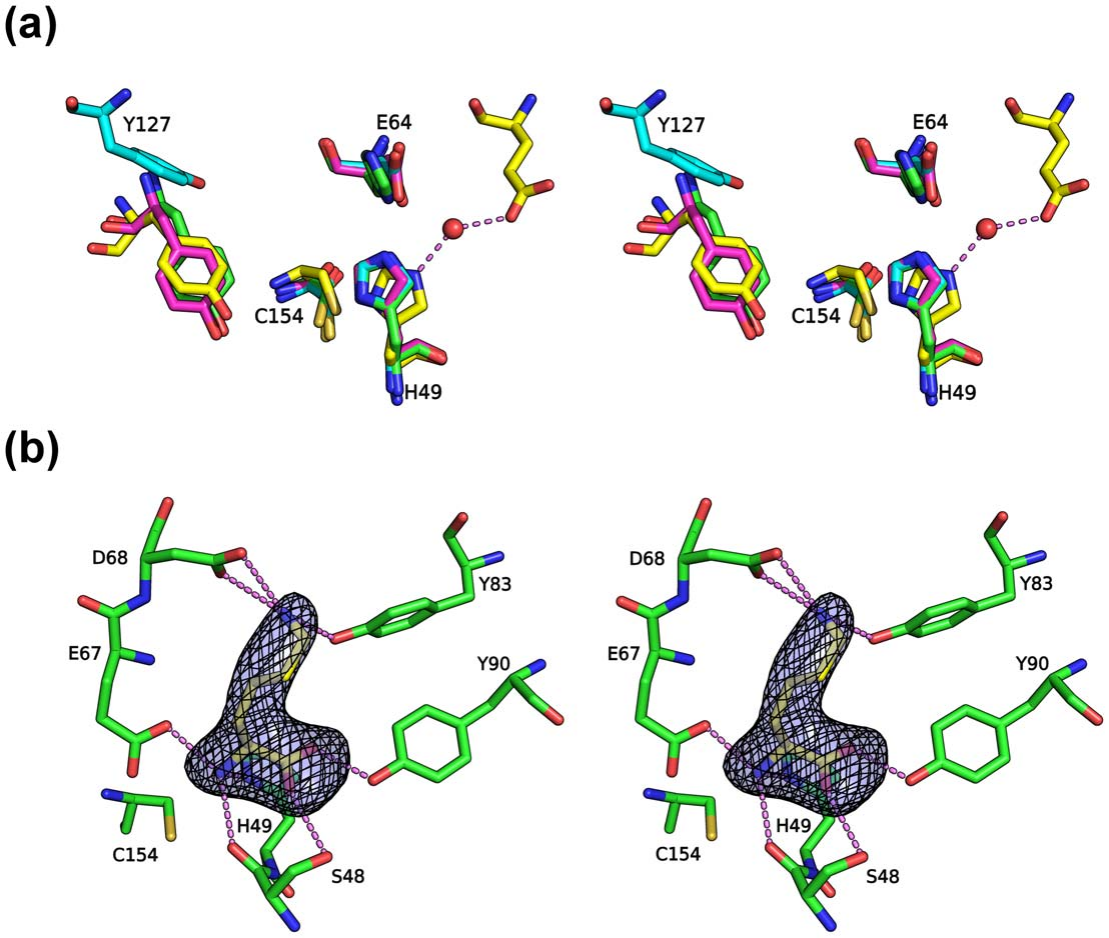
Active site of BcPPNE. (a) Residues essential for the catalytic activity of papain-like NlpC/P60 enzymes: BcYkfC (green; Cys238, His291, His303 and Tyr226), BcPPNE (cyan; Cys154, His49, Glu64 and Tyr127), YiiX (magenta; Cys128, His49, Glu64 and Tyr115) and human PPPDE1 (yellow; Cys108, His38, Glu47, and Tyr101; red; water). Residue numbers are from BcPPNE. (b) Bound lysine identified in the active site. The omit density (Fo-Fc) is contoured at 4.5σ. Hydrogen bonds are shown in dashed lines.

CPNEs contain four catalytic important residues equivalent to those of papain [2], [5]. The first three residues are the catalytic triad consisting of an invariant Cys/His dyad and a polar residue that orients the His imidazole ring that, in turn, deprotonates the catalytic Cys. This polar residue is the acidic Glu164 in BcPPNE, which is also highly conserved in other YaeF/YiiX-family members and in viral PPNEs [1]. The fourth catalytic residue, a conserved tyrosine, appears to be a distinctive signature of NlpC/P60 proteins. It is equivalent to the glutamine (Gln19) of papain, and is likely to interact with the carbonyl group of the P1 residue during catalysis. Tyrosine is an unusual active-site residue in peptidases, although it has been implicated in some metallopeptidases, and is an important substrate binding residue in pepsin and its homologues. Peptidases from families C54 (autophagins) and C78 (UfSP1 and UfSP2 peptidases) of MEROPS database [19] also have a papain-like structure and a tyrosine, instead of the Gln19 of papain [20], [21]. The conformation of the catalytic triad of BcPPNE (Cys154, His49 and Glu64) is identical to those in YiiX and CPNEs, as exemplified in BcYfkC (rmsd ∼0.3 Å for common atoms) (Fig. 3a). The typical position for the third polar residue of the catalytic triad in PPPDE1 is occupied here by a phenylalanine. A nearby water molecule stabilized by Glu47 likely substitutes for this function by providing a hydrogen bond that orients the His38 imidazole (Fig. 3a). Interestingly, mutagenesis studies of LRAT indicated that the third polar residue of the catalytic triad might not be essential for catalysis [8]. Thus, based on the conservation of catalytic residues with previously characterized CPNEs, we conclude that these PPNEs are also cysteine peptidases, or related enzymes. Notably, the position and conformation of Tyr127 in BcPPNE is significantly different when compared to other equivalent residues of proteins in the same family (Fig. 3a), with its hydroxyl group ∼8.5 Å away from the expected location (see below).

Extra, unaccounted-for, electron density was identified at the entrance to the active site of each BcPPNE monomer and was modeled as an L-lysine based on shape and electrostatic complementarity (Fig. 3b). Since L-lysine was not present in any of the protein production or crystallization reagents, it was likely acquired from the expression host. The exact identity of the ligand could not be determined as it could also be another similar amino acid, such as ornithine. The lysine occupies the S1′ subsite and forms a hydrogen-bond network with several conserved protein residues (Ser48, His49, Glu67, Asp68, Tyr83, and Tyr90). Thermofluor experiments indicated that BcPPNE also binds L-arginine and O-phospho-L-serine. We analyzed crystals soaked in presence of 1 mM O-phospho-L-serine. However, O-phospho-L-serine could not be indentified in the electron density maps and L-Lys remained bound (data not shown).

LRAT and G6R both contain these four catalytic residues, supporting a reaction mechanism similar to thiol peptide hydrolysis [22] (Fig. 2c). Additionally, LRAT contains a second cysteine located 7 amino acids downstream of the catalytic cysteine. Both the catalytic and downstream cysteine residues have been shown to be important for the acyltransferase activity of LRAT [23]. This second cysteine corresponds to Cys161 in BcPPNE, which is located on the helix containing the catalytic cysteine C154. However, it is buried and, thus, inaccessible to solvent or substrate. Therefore, in this case, the importance of the second cysteine is likely structural.

### Ligand-induced conformational changes

**Figure 4:**
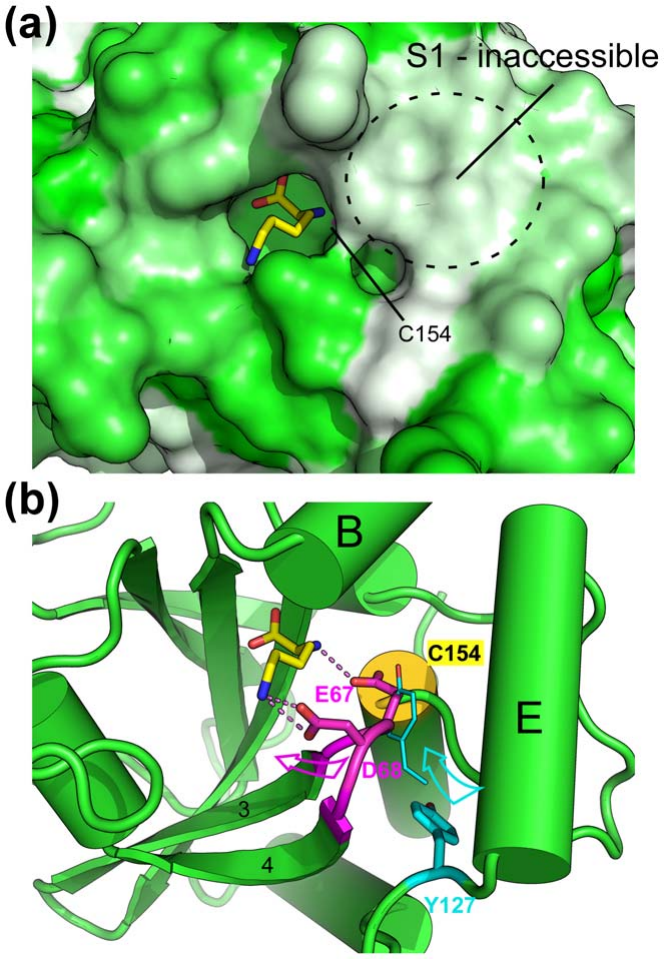
Ligand-induced conformational changes. (a) The active site pocket is occupied by a bound ligand interpreted as a Lys (shown in sticks). The protein surface is colored using a hydrophobicity gradient from white (hydrophobic) to green (hydrophilic). The approximate location of the S-binding pocket is marked by a circle. (b) Interaction between the bound ligand and a nearby loop likely induces structural changes in the active site, which significantly alter the conformation of Tyr127. The expected conformation of this tyrosine during catalysis, derived by analogy to other NlpC/P60 enzymes (Fig. 3a), is shown as thinner sticks. Arrows indicated possible movements needed to restore the productive conformation. Hydrogen bonds are shown as dashed lines.

One striking feature of the active site pocket of BcPPNE is that the tight fit of the binding cavity around the bound lysine at S1′ renders the catalytic cysteine inaccessible to solvent (Fig. 4a), suggesting induced-fit binding. Side chains of Glu67 and Asp68 interact with the free main-chain amine group and the side-chain Nζ of the lysine, respectively. In this conformation, Glu67 occupies the position expected for Tyr127, based on comparison with other NlpC/P60 structures. As a result, the bound lysine seems to have induced the rearrangement of a short loop (residues 66–69), containing Glu67 and Asp68, which, in turn has displaced Tyr127 from its (expected) original location (Fig. 4b). This conformational change may also have induced additional changes near the active site, resulting in the disappearance of the S1 substrate-binding pocket. Thus, we conclude that the ternary complex of BcPPNE may represent a “closed” (or inhibited) conformation of the enzyme.

Crystal packing suggests that the BcPPNE tetramer present in the asymmetric unit (asu) likely represents the biologically relevant species in a dimer of dimers type arrangement. The A/C and B/D dimers are formed through interactions between helices near the active sites, while the A/B and C/D dimer interfaces lie along the outer edges of the central β-sheets. The dimer-dimer interface is mediated by small hydrophobic patches and buries ∼1700 Å^2^ surface area per dimer. Size exclusion chromatography also suggested a weak tetramer in solution, consistent with crystallographic packing. However, the dimer interfaces for A/C and B/D involves regions (near Tyr127) that are suspected to have undergone conformational changes, as described above. Thus, this tetrameric assembly and configuration could also be attributed to ligand binding.

### Identification of a fatty acid in the active site of YiiX

**Figure 5:**
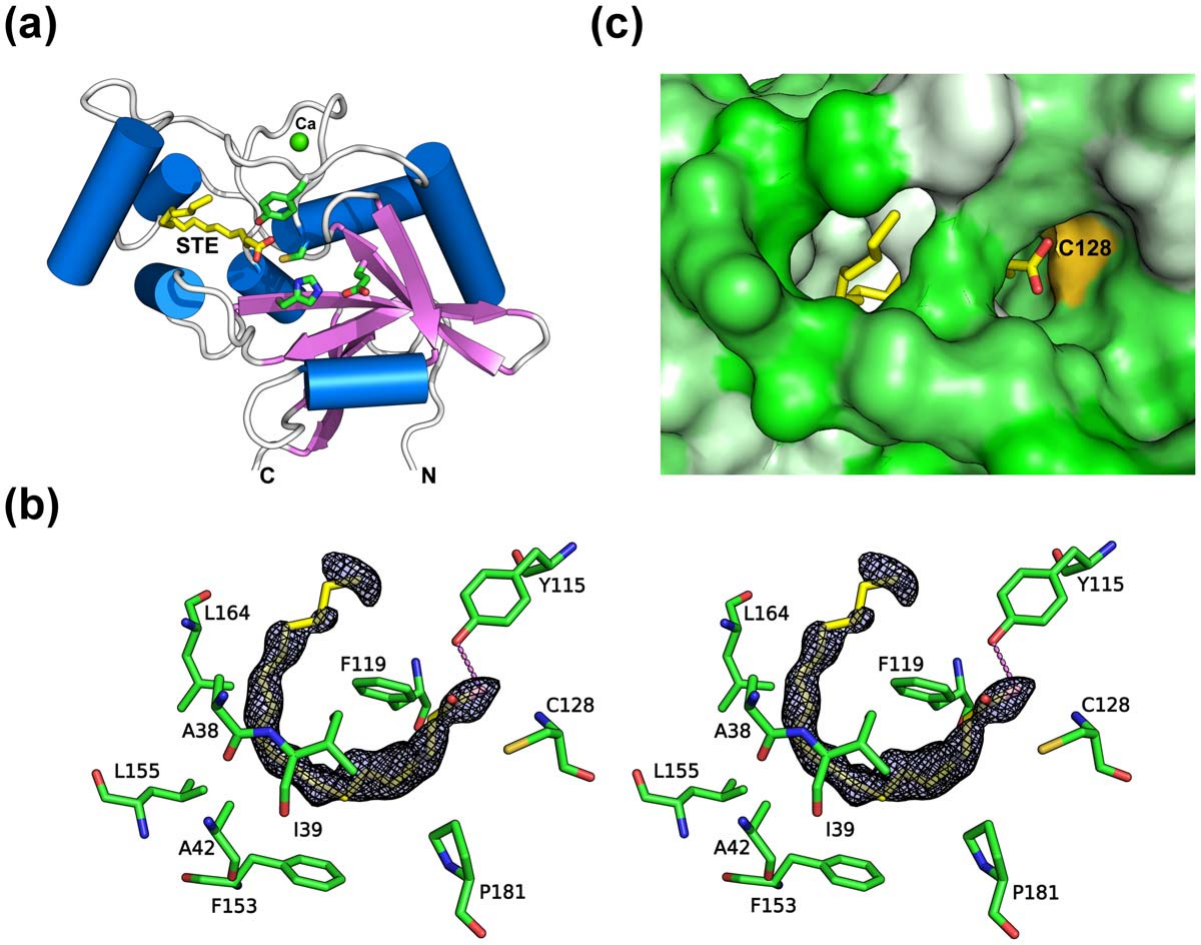
YiiX binds a fatty acid. (a) Ribbon representation of YiiX using our re-refined coordinates of PDB entry 2if6. The bound fatty acid that we interpreted from the electron density and the catalytic residues is shown as sticks. (b) A fatty acid was identified in the active site. The omit density (Fo-Fc) is contoured at 3.0 σ. A hydrogen bond between the ligand and the active site is shown as a dashed line. (c) Surface representation of the fatty acid (shown in sticks) and YiiX (shown as a solvent-accessible surface) interaction. The catalytic cysteine (Cys128) is colored as a gold surface near the carboxyl of the bound fatty acid. The protein surface is colored as a hydrophobicity gradient from green (hydrophilic) to white (hydrophobic).

The YiiX structure was determined previously by NYSGXRC to 1.8 Å resolution (PDB 2if6; Bonanno, J.B., Gilmore, J., Bain, K.T., Powell, A., Ozyurt, S., Wasserman, S., Sauder, J.M., Burley, S.K., and Almo, S.C., unpublished) with two molecules per asu. The electron density suggests that a ligand exists in the S1 pocket of the active site. In order to obtain an improved map to identify the ligand, we re-refined the structure using data from the PDB file using BUSTER-TNT [24] (Fig. 5a). TLS components were refined with one monomer per TLS group. The shape of the density indicated that the ligand is very likely a fatty acid, which we tentatively modeled as a stearic acid (Fig. 5b). Additionally, two calcium ions, one chloride ion and one phosphate were modeled. These modifications improved both the refinement statistics (R_cryst_/R_free_ = 16.5/20.0, compared to starting values of 16.7/21.0) and electron density maps.

The center of the putative U-shaped lipid is buried in a hydrophobic tunnel formed by three helices (Fig. 5a). Both ends of the tunnel are accessible to solvent. The head group of the lipid forms a hydrogen bond with the side chain of Tyr115, which is consistent with the role of this tyrosine in the stabilization of the P1 residue during catalysis. The active site groove of YiiX is accessible to solvent (Fig. 5c). Thus, YiiX represents an “open” conformation of a PPNE.

### A conserved hydrophobic S1 substrate-binding pocket in PPNEs

**Figure 6:**
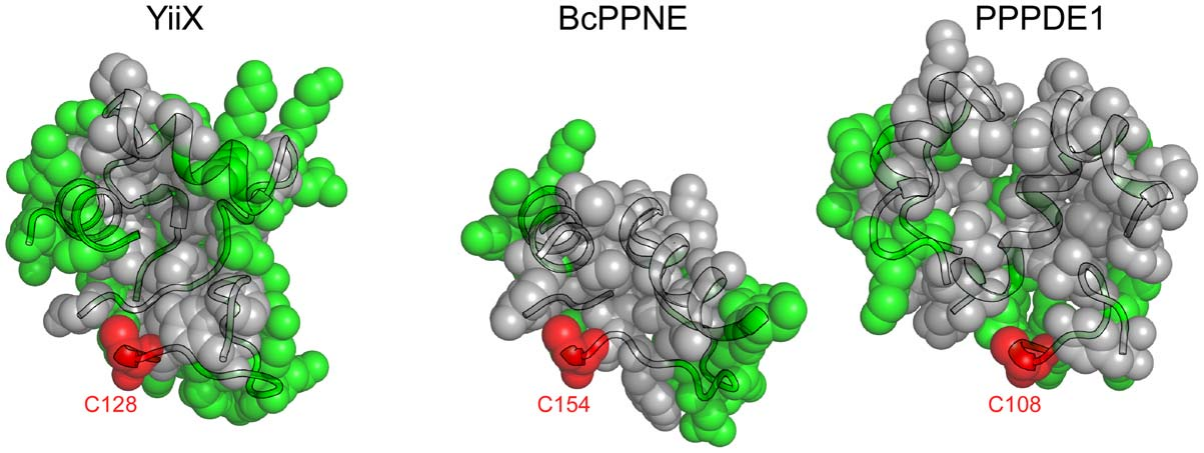
PPNEs have hydrophobic S1 binding pockets. The S1 binding pockets of YiiX, BcPPNE and PPPDE1 are shown as semi-transparent spheres overlaying ribbon representations, with hydrophobic and aromatic residues colored as gray, hydrophilic residues green and the catalytic cysteines in red.

The substrate specificity in CPNEs is defined by loop insertions between conserved secondary structural elements [5]. These insertions produce cavities of different sizes and properties. This observation also appears valid for PPNEs. The S1 substrate-binding pockets of YiiX, BcPPNE and PPPDE1 are formed by insertions between core secondary structural elements, namely loops between β1 and β2, αD and αF, αF and αG, which vary in length and complexity. All three binding pockets are defined by the β1–β2 and αD-αF loops on either sides, and the αF-αG loop at the back. The arrangement of secondary structure elements forming the S1 binding sites is similar in YiiX and PPPDE1. However, the YiiX S1 binding site is narrow and elongated, while PPPDE1 has a much wider pocket (Fig. 6). The loops defining the S1 binding site are simpler in BcPPNE, with the β1–β2 and αD-αF loops each containing a helix (αB and αE). Helix αB is conserved in YiiX and BcPPNE. It is likely the S1 binding site of BcPPNE is defined as a groove between the two helices above, running from the catalytic cysteine toward the N-terminus of αG. Interestingly, all of the S1 binding sites of PPNEs are hydrophobic (Fig. 6).

In contrast, the S′-binding pockets of three PPNEs are all hydrophilic. BcPPNE contains a highly restrictive S1′-site that is formed by the β3–β4 loop, αC and its subsequent 3_10_ helix, as well as the β1-αB loop (Fig. 1). The first two regions are shorter in both YiiX and PPPDE1 (Fig. 2c), which provides more space to potentially accommodate larger substrates in the S′ site.

The conserved hydrophobic nature of PPNEs S1 binding sites differs from that of CPNEs. For example, the BcYkfC binding site contains a conserved Asp-Arg pair and additional polar residues that interact with the peptide substrate [5]. These distinctive binding sites likely indicate their essential role in defining substrate specificity and suggest a different substrate preference for PPNEs.

## Discussion

The PPNE superfamily is emerging as a novel class of proteins of biomedical importance. We provide the first structural report of a bacterial member (BcPPNE), as well as a comparative structural analysis with other PPNEs (*E. coli* YiiX and human PPPDE1) and CPNEs. The structures of the highly divergent NlpC/P60 superfamily of proteins are surprisingly conserved in the following aspects. First, CPNEs and PPNEs share a common core. Second, PPNEs share four important catalytic residues (Cys/His/Tyr and an additional polar residue) that are arranged in the same configuration as in CPNEs. Furthermore, the conserved tyrosine appears to be essential, in addition to the Cys/His dyad [2], [7], [22] in both PPNEs and CPNEs. Unexpectedly, we found that S1 substrate-binding sites of all three PPNEs are hydrophobic, in contrast to their hydrophilic nature in CPNEs. The PPNE structures also sample different ligand-bound and different conformational states, where BcPPNE with an amino acid bound, was in a “closed” conformation, whereas YiiX with a fatty acid bound and *apo*-PPPDE1 were in “open” conformations. Thus, these crystal structures have provided us with a wealth of information about these proteins, even although their specific functions are currently unknown. More importantly, we expect the insights obtained from these structures will also impact the understanding of other proteins in this superfamily.

**Figure 7:**
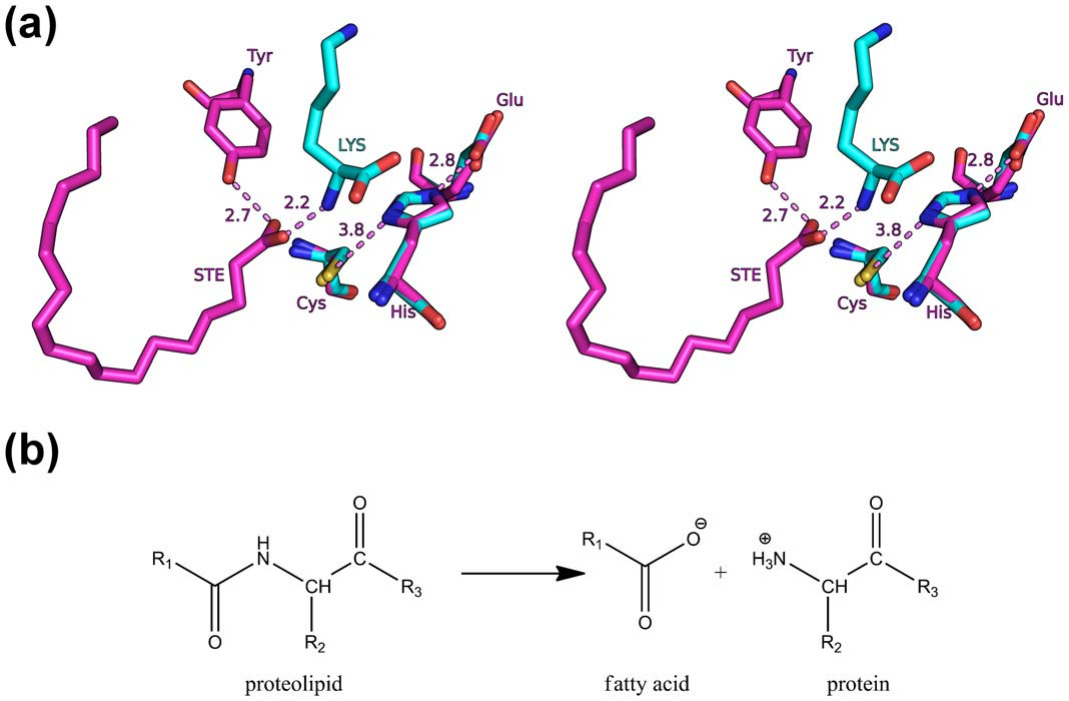
Proposed function for YiiX and BcPPNE. (a) Location of the bound ligands in YiiX and BcPPNE with respect to the conserved catalytic triads. BcPPNE and YiiX were superposed using their catalytic triads. (b) A proposed chemical function for these enzymes where YiiX and BcPPNE cleave the amide linkage between a fatty acid and an amino acid (R1 = polycarbon chain, R2 = side-chain or H, R3 = peptide or OH).

Structures determined by structural genomics often contain endogenous ligands, many of which cannot be unambiguously identified [25]. For proteins of unknown function, the identification of a ligand in the active site can lead to significant insights into the protein function [26]. Interestingly, we observed endogenous compounds in two bacterial PPNEs, bound at different sites in their respective structures (Fig. 7a). Based on the identities of the respective compounds and the position of the scissile bond in the active sites, we suggest that YiiX may function as an amidase by cleaving the amide bond between a fatty acid and a peptide (or protein) (Fig. 7b), such as lipoproteins, N-myristoylated proteins, and lipoamino acids. Alternatively, it could participate in other enzymatic functions involving a fatty acid, for example, palmitoylation of a cysteine. As circular permutation does not change the structure significantly, it is generally assumed the permutated YiiX may be also involved in degradation of the cell wall, as for most CPNEs. However, the narrow and hydrophobic S1 substrate-binding site of YiiX does not appear suitable for binding a peptide. Thus, our proposed substrate specificity for YiiX differs substantially from previous assumptions. BcPPNE may have a similar function as YiiX. However, the substrate for BcPPNE could be simple lipoamino acids, due to the well-defined S1′ site. Fatty acid acylation is a major form of covalent modification that often plays key roles in proteins that regulate cellular structure and function [27]. Our results here provide interesting clues of possible novel means of regulation of these proteins by PPNEs.

The NlpC/P60 superfamily includes several related, but distinct, catalytic functions such as hydrolases, phospholipases and acetyltransferases. Currently, very few such examples are known for cysteine hydrolase families, except for guanine 5′-monophosphate synthetase, carbamoyl-phosphate synthase and dihydroorotase, which are all homologues of peptidases in family C26 (gamma-glutamyl hydrolase). Serine hydrolases, on the other hand, commonly have different enzymatic functions within a family; for example, lipases and cholinesterases are closely related to peptidases in family S9, and lactamases are related to the D-Ala-D-Ala carboxypeptidases in family S12.

Our studies of the canonical P60-like family suggest that the active site is most conserved at the S1 site where it facilitates recognition of γ-D-Glu [5]. Structural analysis of PPNEs also suggests that the S1 binding sites are selective for specific substrates. Specificity at for the P1 residue in NlpC/P60 superfamily likely arises from the requirement of placing the P1 carbonyl group next to the hydroxyl group of the conserved tyrosine during catalysis.

Activities of eukaryotic PPNEs LRAT and H-Rev107 are related to lipid-containing substrates. LRAT converts all-trans-retinol into all-trans-retinyl esters [28], while H-Rev107 functions as a thiol hydrolase-type phospholipase A1/2 [13]. These eukaryotic PPNEs may have evolved from bacterial YaeF/YiiX-like PPNEs through horizontal gene transfer [1]. It is plausible that their substrate specificity for a lipid-like substrate may have been acquired from the bacterial ancestor. We have shown here that a bacterial PPNE YiiX can, indeed, bind lipid. Thus, the specificity for lipids by PPNEs could initially arise from a common bacterial ancestor, for example, as a result of a circular permutation event.

Furthermore, the molecular architectures of PPNEs suggest that their functions are likely associated with the membrane. Many PPNEs are fused to membrane targeting modules, such as lipoprotein signal peptides, the PH domain, and the C2 domain. The regulation of vulval cell morphogenesis by EGL-26 in *Caenorhabditis elegans* is dependent on the membrane localization of the PPNE domain [9]. The peptidase or related activities of PPNEs could also be involved in the regulation of fatty acylated proteins in viruses. Viral PPNEs are also likely to have evolved from a bacterial ancestor [6], and may also retain the ability to bind lipid. It is well known that myristoylated and palmitoylated proteins are important factors in the virus life cycle [27]. For example, multiple fatty acylated proteins were identified in vaccinia virus [29], and could be potentially regulated by G6R. A lipid-peptide linkage usually targets the attached protein to the membrane [27]. As a result, the peptidase-related function of PPNEs from virus may be needed for the maturation of the viral particle [1], [6]. For these reasons, we speculate that PPPDE1 could bind a more complex lipid moiety in its larger, hydrophobic, S1 substrate-binding pocket.

In summary, we present evidence that PPNEs are a unique class of enzymes with a unique hydrophobic S1 substrate-binding pocket, which are likely specific for lipid-like substrates. As a result, the activities of PPNEs may be related to the metabolism of lipids or lipidified proteins. The information presented here, in combination with further biochemical and biophysical studies, will yield valuable insights into the functional role of these interesting proteins.

## Materials and Methods

### Cloning and protein production

Clones were generated using the Polymerase Incomplete Primer Extension (PIPE) cloning method [30]. The gene encoding BcPPNE (GenBank: NP_982244, UniProt: Q74NK7, locus name: BCE_A0238) was amplified by polymerase chain reaction (PCR) from *B. cereus* NRS248 genomic DNA using *PfuTurbo* DNA polymerase (Stratagene) and I-PIPE (Insert) primers (forward primer, 5′-ctgtacttccagggcATGGGAACAGATAAATTTAATAACA-3′; reverse primer, 5′-aattaagtcgcgttaTTCTATCTGCGCAATAGGAAGAACATG-3′, target sequence in upper case) that included sequences for the predicted 5′ and 3′ ends. The expression vector, pSpeedET, which encodes an amino-terminal tobacco etch virus (TEV) protease-cleavable expression and purification tag (MGSDKIHHHHHHENLYFQ/G), was PCR amplified with V-PIPE (Vector) primers (forward primer: 5′-taacgcgacttaattaactcgtttaaacggtctccagc-3′, reverse primer: 5′-gccctggaagtacaggttttcgtgatgatgatgatgatg-3′). V-PIPE and I-PIPE PCR products were mixed to anneal the amplified DNA fragments together. *E. coli* GeneHogs (Invitrogen) competent cells were transformed with the I-PIPE/V-PIPE mixture and dispensed on selective LB-agar plates. The cloning junctions were confirmed by DNA sequencing. Expression was performed in a selenomethionine-containing medium at 37°C. Selenomethionine was incorporated via inhibition of methionine biosynthesis [31], which does not require a methionine auxotrophic strain. At the end of fermentation, lysozyme was added to the culture to a final concentration of 250 µg/ml, and the cells were harvested and frozen. After one freeze/thaw cycle, the cells were homogenized in lysis buffer [50 mM HEPES pH 8.0, 50 mM NaCl, 10 mM imidazole, and 1 mM Tris(2-carboxyethyl)phosphine-HCl (TCEP)] and passed through a Microfluidizer (Microfluidics). The lysate was clarified by centrifugation at 32,500×g for 30 minutes and loaded onto a nickel-chelating resin (GE Healthcare) pre-equilibrated with lysis buffer, the resin washed with wash buffer [50 mM HEPES pH 8.0, 300 mM NaCl, 40 mM imidazole, 10% (v/v) glycerol, and 1 mM TCEP], and the protein was eluted with elution buffer [20 mM HEPES pH 8.0, 300 mM imidazole, 10% (v/v) glycerol, and 1 mM TCEP]. The eluate was buffer exchanged with HEPES crystallization buffer [20 mM HEPES pH 8.0, 200 mM NaCl, 40 mM imidazole, and 1 mM TCEP] using a PD-10 column (GE Healthcare) and concentrated to 9.6 mg/ml by centrifugal ultrafiltration (Millipore). The oligomeric state of BcPPNE in solution was determined using a 1×30 cm^2^ Superdex 200 size exclusion column (GE Healthcare) coupled with miniDAWN (Wyatt Technology) static light scattering (SEC/SLS) and Optilab differential refractive index detectors (Wyatt Technology). The mobile phase consisted of 20 mM Tris pH 8.0, 150 mM NaCl, and 0.02% (w/v) sodium azide. The molecular weight was calculated using ASTRA 5.1.5 software (Wyatt Technology).

### Crystallization and diffraction screening

BcPPNE was crystallized using the nanodroplet vapor diffusion method [32] with standard JCSG crystallization protocols [15]. Sitting drops composed of 200 nl protein solution mixed with 200 nl crystallization solution in a sitting drop format were equilibrated against a 50 µl reservoir at 277 K for 18 days prior to harvest. The crystallization reagent consisted of 0.8 M KH_2_PO_4_, 0.8 M NaH_2_PO_4_, and 0.1 M HEPES pH 7.5. Glycerol was added to a final concentration of 20% (v/v) as a cryoprotectant. Initial screening for diffraction was carried out using the Stanford Automated Mounting system (SAM) [33] at the Stanford Synchrotron Radiation Lightsource (SSRL, Menlo Park, CA).

### Data collection, structure solution, and refinement

Single-wavelength anomalous diffraction (SAD) data were collected at wavelength corresponding to the peak of a selenium MAD experiment at 100 K using Mar CCD 300 detector (Rayonix) at APS beamline GM/CA 23-ID-D. Due to the long *c*-axis, a small oscillation angle of 0.1 degrees was used in order to avoid overlaps. The data were integrated and reduced using XDS and then scaled with the program XSCALE [34]. 22 Selenium sites were located with SHELXD [35]. Phase refinement, density modification and automatic model building were performed using autoSHARP [36] (Figure of merit 0.21) and RESOLVE [37]. Since the initial phases were poor, the 4-fold non-crystallographic operators derived from heavy atom positions were used to improve map quality through NCS averaging. Automated process by RESOLVE produced an initial model that was 78% complete. Further model completion and refinement were performed manually with COOT [38] and REFMAC [39] of the CCP4 suite [40]. TLS parameters were refined with each monomer as a rigid body group. Tight, non-crystallographic symmetry between monomers (rmsd 0.05 Å for main chain, 0.1 Å for side chains for regions that are highly similar: residues 4–58, 60–145, 150–195), as well as experimental phases, were used as restraints during refinement due to the moderate resolution. Data and refinement statistics are summarized in Table 1. Analysis of the stereochemical quality of the model was accomplished using MolProbity [18]. All molecular graphics were prepared with PyMOL (http://www.pymol.org). Atomic coordinates and experimental structure factors for BcPPNE at 2.5 Å resolution have been deposited in the PDB under accession code 3kw0.
